# Royal Medical and Chirurgical Society

**Published:** 1897-04-17

**Authors:** 


### Medical Progress and Hospital Clinics.

[*The Editor will be glad to receive offers of co-operation and contributions from members of the profession. All letters should be addressed to* The Editor, at the Office, 28 & 29, Southampton Street, Strand, London, W.C.]

### ROYAL MEDICAL AND CHIRURGICAL SOCIETY.

At the meeting held on April 13th a paper was read by Mr. Lawford Knaggs on a case of compound depressed fracture of the skull, with cerebral abscess and hernia cerebri, in which recovery took place. He then entered into a full consideration of the subject of hernia cerebri based on 109 collected cases. The intracranial conditions which produced this condition were (1) increased tension, (2) a softened or diffluent state of a portion of the cerebral substance, (3) inflammation of the brain substance; which led to an increase in its bulk, to softening, and, when prolonged, to its replacement by granulation tissue, and finally by cicatrix. The causes were traumatism and sepsis. In regard to treatment, non-interference should be the rule. Rigid cleanliness should be observed, and the tumour allowed to run its own course. Operation, however, might be necessary if abscess were present; but it should aim at evacuation and not at mutilation. Apart from abscess, the necessity for operation was extremely rare; and, now that the presence of embedded fragments could be diagnosed by skiagraphy, it was difficult to imagine a condition in which excision could be justified.

